# Ab initio predictions link the neutron skin of ^208^Pb to nuclear forces

**DOI:** 10.1038/s41567-022-01715-8

**Published:** 2022-08-22

**Authors:** Baishan Hu, Weiguang Jiang, Takayuki Miyagi, Zhonghao Sun, Andreas Ekström, Christian Forssén, Gaute Hagen, Jason D. Holt, Thomas Papenbrock, S. Ragnar Stroberg, Ian Vernon

**Affiliations:** 1 TRIUMF, Vancouver, British Columbia Canada; 2 Department of Physics, Chalmers University of Technology, Gothenburg, Sweden; 3 Department of Physics, Technische Universität Darmstadt, Darmstadt, Germany; 4 ExtreMe Matter Institute EMMI, GSI Helmholtzzentrum für Schwerionenforschung GmbH, Darmstadt, Germany; 5 Department of Physics and Astronomy, University of Tennessee, Knoxville, TN USA; 6 Physics Division, Oak Ridge National Laboratory, Oak Ridge, TN USA; 7 Department of Physics, McGill University, Montreal, Quebec Canada; 8 Department of Physics, University of Washington, Seattle, WA USA; 9 Physics Division, Argonne National Laboratory, Lemont, IL USA; 10 Department of Mathematical Sciences, Durham University, Durham, UK

**Keywords:** Theoretical nuclear physics, Astronomy and astrophysics

## Abstract

Heavy atomic nuclei have an excess of neutrons over protons, which leads to the formation of a neutron skin whose thickness is sensitive to details of the nuclear force. This links atomic nuclei to properties of neutron stars, thereby relating objects that differ in size by orders of magnitude. The nucleus ^208^Pb is of particular interest because it exhibits a simple structure and is experimentally accessible. However, computing such a heavy nucleus has been out of reach for ab initio theory. By combining advances in quantum many-body methods, statistical tools and emulator technology, we make quantitative predictions for the properties of ^208^Pb starting from nuclear forces that are consistent with symmetries of low-energy quantum chromodynamics. We explore 10^9^ different nuclear force parameterizations via history matching, confront them with data in select light nuclei and arrive at an importance-weighted ensemble of interactions. We accurately reproduce bulk properties of ^208^Pb and determine the neutron skin thickness, which is smaller and more precise than a recent extraction from parity-violating electron scattering but in agreement with other experimental probes. This work demonstrates how realistic two- and three-nucleon forces act in a heavy nucleus and allows us to make quantitative predictions across the nuclear landscape.

## Abstract (web summary)

Predictions of the properties of ^208^Pb from first principles augmented by statistical learning techniques reproduce those seen in experiments but rule out very thick neutron skins.

## Main

Neutron stars are extreme astrophysical objects whose interiors may contain exotic new forms of matter. The structure and size of neutron stars are linked to the thickness of the neutron skin in atomic nuclei via the neutron-matter equation of state^1–3^. The nucleus ^208^Pb is an attractive target for exploring this link in both experimental^4,5^ and theoretical^2,6,7^ studies owing to the large excess of neutrons and its simple structure. Mean-field calculations predict a wide range for *R*_skin_(^208^Pb) because the isovector parts of nuclear energy density functionals are not well constrained by binding energies and charge radii^2,7–9^. Additional constraints may be obtained^10^ by including the electric dipole polarizability of ^208^Pb, though this comes with a model dependence^11^ which is difficult to quantify. In general, the estimation of systematic theoretical uncertainties is a challenge for mean-field theory.

**Fig. 1:**
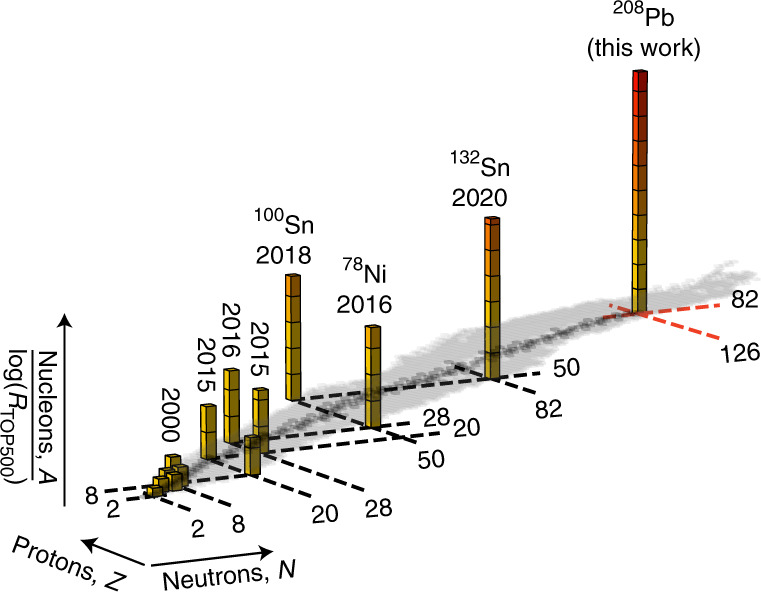
Trend of realistic ab initio computations for the nuclear *A*-body problem. The bars highlight the years of the first realistic computations of doubly magic nuclei. The height of each bar corresponds to the mass number *A* divided by the logarithm of the total compute power *R*_TOP500_ (in flops s^−1^) of the pertinent TOP500 list^45^. This ratio would be approximately constant if progress were solely due to exponentially increasing computing power. However, algorithms which instead scale polynomially in *A* have greatly increased the reach. Source data

In contrast, precise ab initio computations, which provide a path to comprehensive uncertainty estimation, have been accomplished for the neutron-matter equation of state^12–14^ and the neutron skin in the medium-mass nucleus ^48^Ca (ref. ^15^). However, up to now, treating ^208^Pb within the same framework was out of reach. Owing to breakthrough developments in quantum many-body methods, such computations are now becoming feasible for heavy nuclei^16–19^. The ab initio computation of ^208^Pb we report herein represents a significant step in mass number from the previously computed tin isotopes^16,17^ (Fig. 1). The complementary statistical analysis in this work is enabled by emulators (for mass number *A* ≤ 16) which mimic the outputs of many-body solvers but are orders of magnitude faster.

In this paper, we develop a unified ab initio framework to link the physics of nucleon–nucleon scattering and few-nucleon systems to properties of medium- and heavy-mass nuclei up to ^208^Pb, and ultimately to the nuclear-matter equation of state near saturation density.

## Linking models to reality

Our approach to constructing nuclear interactions is based on chiral effective field theory (EFT)^20–22^. In this theory, the long-range part of the strong nuclear force is known and stems from pion exchanges, while the unknown short-range contributions are represented as contact interactions; we also include the Δ isobar degree of freedom^23^. At next-to-next-to leading order in Weinberg’s power counting, the four pion–nucleon low-energy constants (LECs) are tightly fixed from pion–nucleon scattering data^24^. The 13 additional LECs in the nuclear potential must be constrained from data.

**Extended Data Fig. 1:**
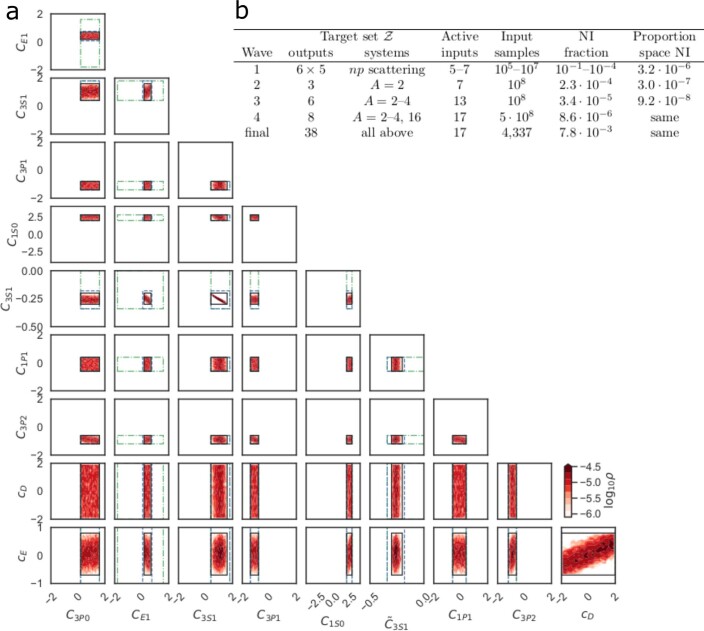
History-matching waves. **a**, The initial parameter domain used at the start of history-matching wave 1 is represented by the axes limits for all panels. This domain is iteratively reduced and the input volumes of waves 2, 3, and 4 are indicated by green/dash-dotted, blue/dashed, black/solid rectangles. The logarithm of the optical depths ${\log }_{10}\rho$ (indicating the density of non-implausible samples in the final wave) are shown in red with darker regions corresponding to a denser distribution of non-implausible samples. **b**, Four waves of history matching were used in this work plus a fifth one to refine the final set of non-implausible samples. The neutron-proton scattering targets correspond to phase shifts at six energies (*T*_lab_ = 1, 5, 25, 50, 100, 200 MeV) per partial wave: ^1^*S*_0_, ^3^*S*_1_, ^1^*P*_1_, ^3^*P*_0_, ^3^*P*_1_, ^3^*P*_2_. The *A* = 2 observables are *E*(^2^H), *R*_p_(^2^H), *Q*(^2^H), while *A* = 3, 4 are *E*(^3^H), *E*(^4^He), *R*_p_(^4^He). Finally, *A* = 16 targets are *E*(^16^O), *R*_p_(^16^O). The number of active input parameters is indicated in the fourth column. The number of inputs sets being explored, and the fraction of non-implausible samples that survive the imposed implausibility cutoff(s) are shown in the fifth and sixth columns, respectively. Finally, the proportion of the parameter space deemed non implausible is listed in the last column. Note that no additional reduction of the non-implausible domain is achieved in the fourth and final waves, in which ^16^O observables are included, but that parameter correlations are enhanced. Source data

**Extended Data Fig. 2:**
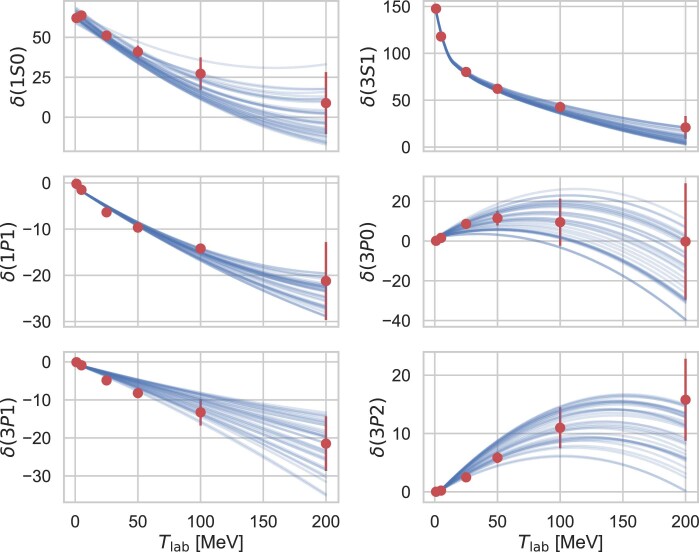
Neutron-proton scattering phase shifts. 34 interaction samples survive the final implausibility cutoff with respect to neutron-proton phase shifts *δ* in *S* and *P* waves up to 200 MeV. The red circles are from the Granada phase shift analysis^78^, while the 2*σ* error bars are dominated by the estimated EFT truncation errors^64^.

We use history matching^25,26^ to explore the modelling capabilities of ab initio methods by identifying a non-implausible region in the vast parameter space of LECs, for which the model output yields acceptable agreement with selected low-energy experimental data (denoted herein as history-matching observables). The key to efficiently analyse this high-dimensional parameter space is the use of emulators based on eigenvector continuation^27–29^ that accurately mimic the outputs of the ab initio methods but at several orders of magnitude lower computational cost. We consider the following history-matching observables: nucleon–nucleon scattering phase shifts up to an energy of 200 MeV; the energy, radius and quadrupole moment of ^2^H; and the energies and radii of ^3^H, ^4^He and ^16^O. We perform five waves of this global parameter search (Extended Data Figs. 1 and 2), sequentially ruling out implausible LECs that yield model predictions too far from experimental data. For this purpose, we use an implausibility measure (Methods) that links our model predictions and experimental observations as

1 $$z=M(\theta )+{\varepsilon }_{\exp }+{\varepsilon }_{{{{\rm{em}}}}}+{\varepsilon }_{{{{\rm{method}}}}}+{\varepsilon }_{{{{\rm{model}}}}},$$

relating the experimental observations *z* to emulated ab initio predictions *M*(*θ*) via the random variables ${\varepsilon }_{\exp }$, *ε*_em_, *ε*_method_ and *ε*_model_ that represent experimental uncertainties, the emulator precision, method approximation errors and the model discrepancy due to the EFT truncation at next-to-next-to leading order, respectively. The parameter vector *θ* corresponds to the 17 LECs at this order. The method error represents, for example, model space truncations and other approximations in the employed ab initio many-body solvers. The model discrepancy *ε*_model_ can be specified probabilistically since we assume to operate with an order-by-order improvable EFT description of the nuclear interaction (see Methods for details).

The final result of the five history-matching waves is a set of 34 non-implausible samples in the 17-dimensional parameter space of the LECs. We then perform ab initio calculations for nuclear observables in ^48^Ca and ^208^Pb, as well as for properties of infinite nuclear matter.

## Ab initio computations of ^208^Pb

**Extended Data Fig. 3:**
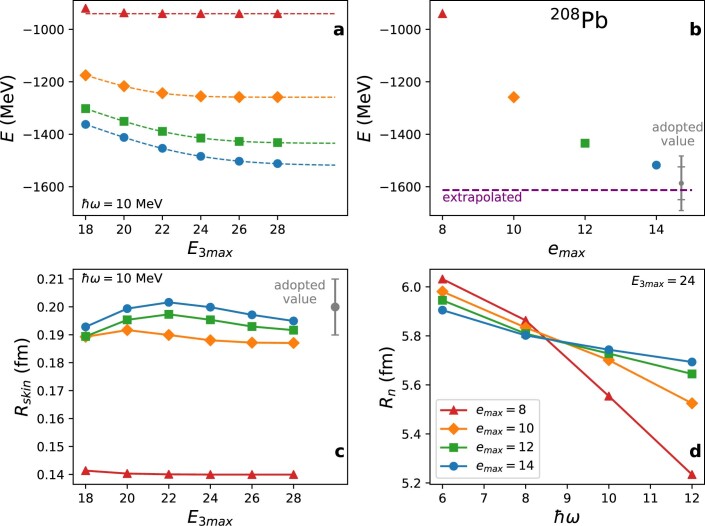
Convergence of energy and radius observables of ^208^Pb with the ${e}_{\max }$ and ${E}_{{{{\rm{3max}}}}}$ truncations. **a**, Ground state energy as a function of ${E}_{{{{\rm{3max}}}}}$. The dashed lines indicate a Gaussian fit. **b**, Ground state energy (extrapolated in ${E}_{{{{\rm{3max}}}}}$ as a function of ${e}_{\max }$. The smaller error bar on the adopted value indicate the error due to model space extrapolation, and the larger error bar also includes the method uncertainty. **c**, Neutron skin as a function of ${E}_{{{{\rm{3max}}}}}$. **d**, Neutron radius as a function of oscillator basis frequency *ℏω* for a series of ${e}_{\max }$ cuts.

**Extended Data Fig. 4:**
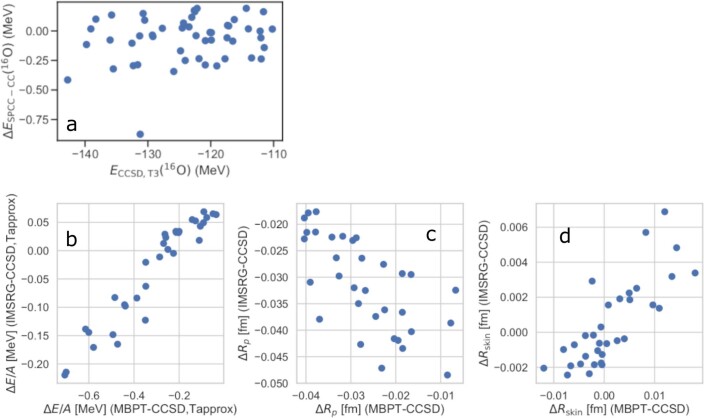
Precision of sub-space coupled-cluster emulator and many-body solvers. **a**, Cross-validation of the SP-CCSDT-3 emulator for the ground-state energy of ^16^O. Results from full computations using CCSDT-3 are compared with emulator predictions for 50 samples from the 17 dimensional space of LECs. The standard deviation for the residuals ΔE_SPCC-CC_ is 0.19 MeV. **b,c,d**, Differences between IMSRG and CC results versus differences between MBPT and CC results for the ground-state energy per nucleon ΔE/A (panel b), the point-proton radius ΔR_p_ (panel c), and the neutron-skin ΔR_skin_ (panel d) of ^208^Pb using the 34 non-implausible interactions obtained from history matching (see text for more details). The CC results for the ground-state energy include approximate triples corrections.

We employ the coupled-cluster (CC)^12,30,31^, in-medium similarity renormalization group (IMSRG)^32^ and many-body perturbation theory (MBPT) methods to approximately solve the Schrödinger equation and obtain the ground-state energy and nucleon densities of ^48^Ca and ^208^Pb. We analyse the model space convergence and use the differences between the CC, IMSRG and MBPT results to estimate the method approximation errors (Methods and Extended Data Figs. 3 and 4). The computational cost of these methods scales (only) polynomially with increasing numbers of nucleons and single-particle orbitals. The main challenge in computing ^208^Pb is the vast number of matrix elements of the three-nucleon (3N) force which must be handled. We overcome this limitation by using a recently introduced storage scheme in which we only store linear combinations of matrix elements directly entering the normal-ordered two-body approximation^19^ (see Methods for details).

**Fig. 2:**
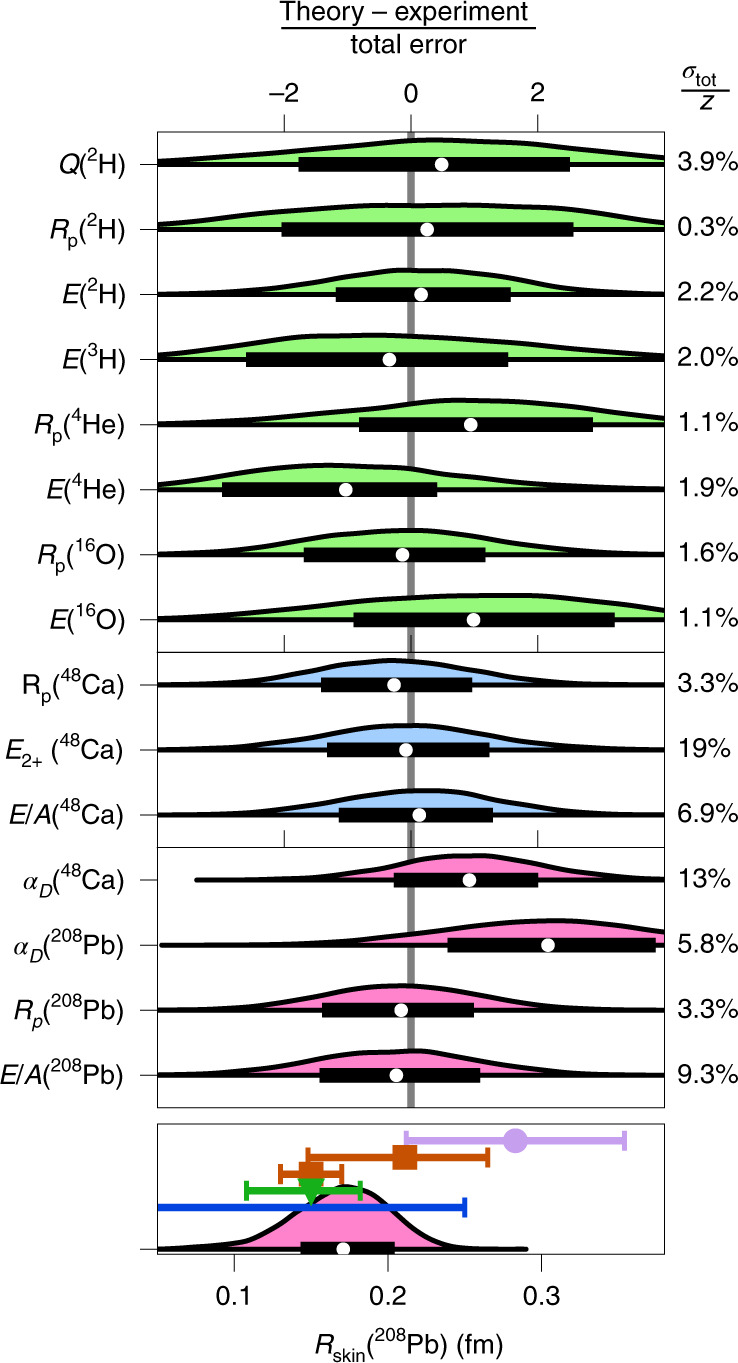
Ab initio posterior predictive distributions for light to heavy nuclei. Model checking is indicated by green (blue) distributions, corresponding to observables used for history-matching (likelihood calibration), while pure predictions are shown as pink distributions. The nuclear observables shown are the quadrupole moment *Q*, point-proton radii *R*_p_, ground-state energies *E* (or energy per nucleon *E*/*A*), 2^+^ excitation energy ${E}_{{2}^{+}}$ and electric dipole polarizabilities *α*_D_. See Extended Data Table 1 for the numerical specification of the experimental data (*z*), errors (*σ*_*i*_), medians (white circle) and 68% credibility regions (thick bar). The prediction for *R*_skin_(^208^Pb) in the bottom panel is shown on an absolute scale and compared with experimental results using electroweak^5^ (purple), hadronic^34,35^ (red), electromagnetic^4^ (green) and gravitational wave^36^ (blue) probes (from top to bottom; see Extended Data Fig. 7b for details). Source data

**Extended Data Fig. 5:**
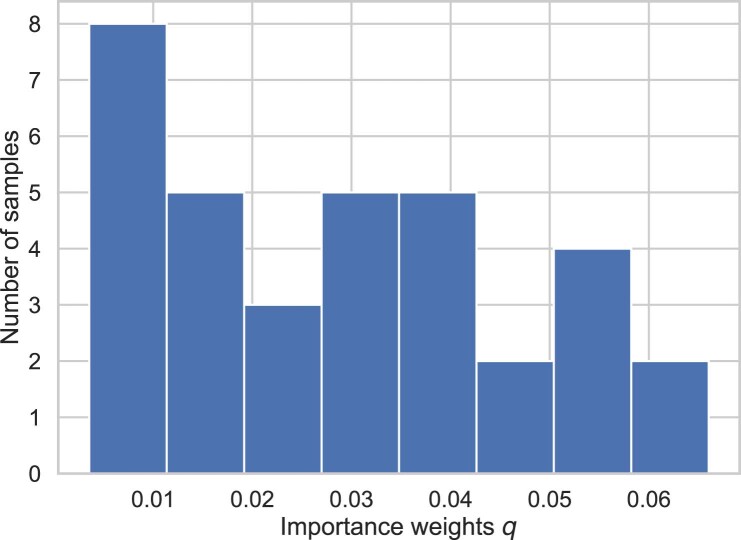
Importance weights. Histogram of importance weights for the 34 non-implausible interaction samples. These are obtained from likelihood calibration as defined in Eq. (6).

Our ab initio predictions for finite nuclei are summarized in Fig. 2. The statistical approach that leads to these results is composed of three stages. First, history matching identified a set of 34 non-implausible interaction parameterizations. Second, model calibration is performed by weighting these parameterizations—serving as prior samples—using a likelihood measure according to the principles of sampling/importance resampling^33^. This yields 34 weighted samples from the LEC posterior probability density function (Extended Data Fig. 5). Specifically, we assume independent EFT and many-body method errors and construct a normally distributed data likelihood encompassing the ground-state energy per nucleon *E*/*A* and the point-proton radius *R*_p_ for ^48^Ca, and the energy ${E}_{{2}^{+}}$ of its first excited 2^+^ state. Our final predictions are therefore conditional on this calibration data.

We have tested the sensitivity of final results to the likelihood definition by repeating the calibration with a non-diagonal covariance matrix or a Student *t* distribution with heavier tails, finding small (~1%) differences in the predicted credible regions. The EFT truncation errors are quantified by studying ab initio predictions at different orders in the power counting for ^48^Ca and infinite nuclear matter. We validate our ab initio model and error assignments by computing the posterior predictive distributions, including all relevant sources of uncertainty, for both the replicated calibration data (blue) and the history-matching observables (green) (Fig. 2). The percentage ratios *σ*_tot_/*z* of the (theory-dominated) total uncertainty to the experimental value are given in the right margin.

Finally, having built confidence in our ab initio model and underlying assumptions, we predict *R*_skin_(^208^Pb), *E*/*A* and *R*_p_ for ^208^Pb, *α*_D_ for ^48^Ca and ^208^Pb as well as nuclear matter properties, by employing importance resampling^33^. The corresponding posterior predictive distributions for ^48^Ca and ^208^Pb observables are shown in Fig. 2 (lower panels, pink). Our prediction *R*_skin_(^208^Pb) = 0.14–0.20 fm exhibits a mild tension with the value extracted from the recent parity-violating electron scattering experiment PREX^5^ but is consistent with the skin thickness extracted from elastic proton scattering^34^, antiprotonic atoms^35^ and coherent pion photoproduction^4^ as well as constraints from gravitational waves from merging neutron stars^36^.

**Fig. 3:**
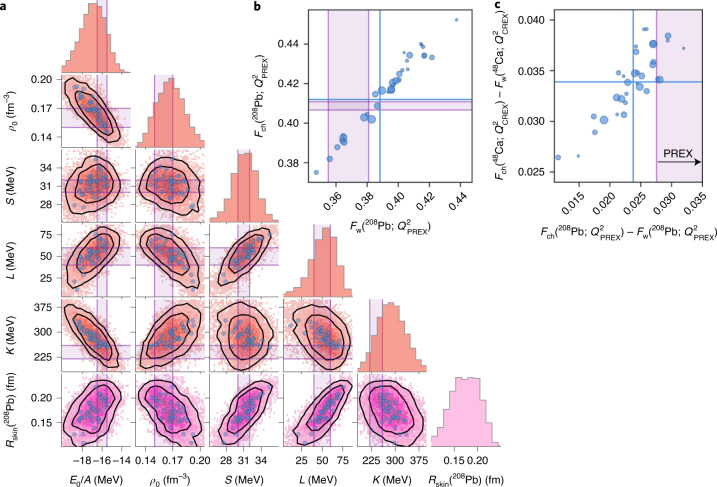
Posterior predictive distribution for *R*_skin_(^208^Pb) and nuclear matter at saturation density. **a**, Predictions for the saturation energy per particle *E*_0_/*A* and density *ρ*_0_ of symmetric nuclear matter, its compressibility *K*, the symmetry energy *S* and its slope *L* are correlated with the those for *R*_skin_(^208^Pb). The sampled bivariate distributions (pink/orange squares) are shown with 68% and 90% credible regions (black lines) and a scatter plot of the predictions with the 34 non-implausible samples before error sampling (blue dots). Empirical nuclear-matter properties are indicated by purple bands (Extended Data Table 2). **b**, Predictions with the 34 non-implausible samples for the electric *F*_ch_ versus weak *F*_w_ charge form factors for ^208^Pb at the momentum transfer considered in the PREX experiment^5^. **c**, The difference between the electric and weak charge form factors for ^48^Ca and ^208^Pb at the momentum transfers *Q*_CREX_ = 0.873 fm^−1^ and *Q*_PREX_ = 0.3978 fm^−1^ that are relevant for the CREX and PREX experiments, respectively. Experimental data (purple bands) in **b** and **c** are from ref. ^5^, the size of the markers indicates the importance weight and blue lines correspond to weighted means. Source data

We also compute the weak form factor *F*_w_(*Q*^2^) at momentum transfer *Q*_PREX_ = 0.3978(16) fm^−1^, which is more directly related to the parity-violating asymmetry measured in the PREX experiment. We observe a strong correlation with the more precisely measured electric charge form factor *F*_ch_(*Q*^2^) (Fig. 3b). While we have not quantified the EFT and method errors for these observables, we find a small variance among the 34 non-implausible predictions for the difference *F*_w_(*Q*^2^) − *F*_ch_(*Q*^2^) for both ^48^Ca and ^208^Pb (Fig. 3c).

## Ab initio computations of infinite nuclear matter

**Extended Data Fig. 6:**
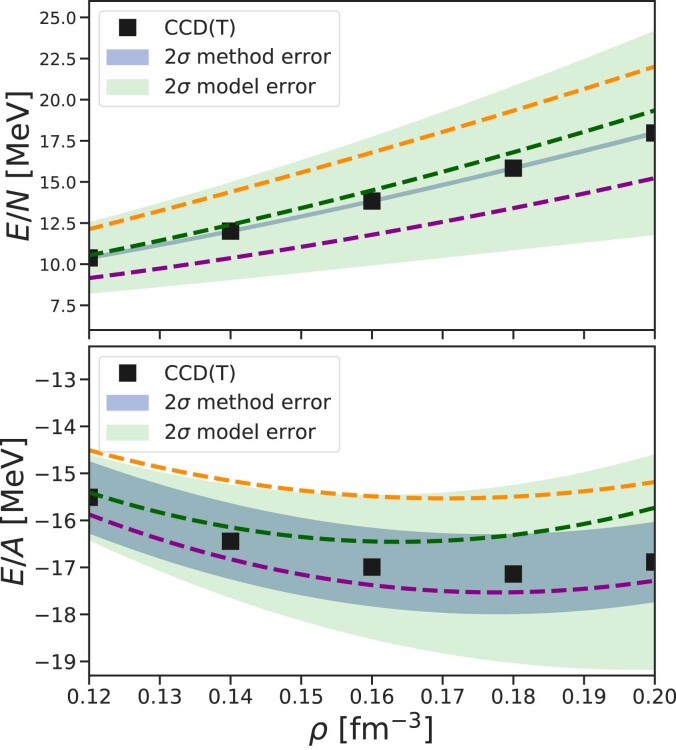
Bayesian machine learning error model. The equation of state of pure neutron matter (top) and symmetric nuclear matter (bottom) calculated with CCD(T) (black squares) are shown along with the corresponding method error (blue shade) and EFT truncation error (green shade) for one representative interaction. Errors are correlated as a function of density *ρ* and the dashed orange, green and purple curves illustrate predictions with randomly sampled method and model errors drawn from the respective multitask Gaussian processes. Correlations extend between pure neutron matter (*E*/*N*) and symmetric nuclear matter (*E*/*A*) energies per particle which is represented here by curves in the same colour. Note that the method error is very small in neutron matter due to the small finite-size effect and the small differences between CCD and CCD(T) results (the Pauli principle prevents short-ranged three-neutron correlations).

**Extended Data Fig. 7:**
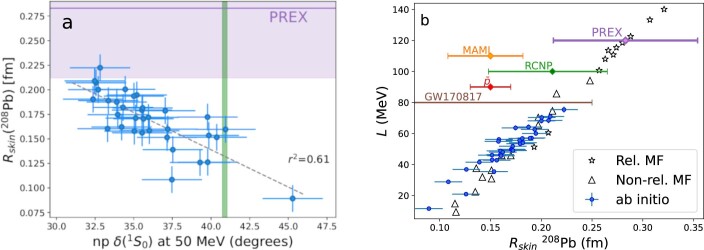
Correlation of *R*_skin_( ^208^Pb) with scattering data and ***L***. **a** Correlation of computed *R*_skin_( ^208^Pb) with the proton-neutron ^1^S_0_ phase shift *δ*(^1^S_0_) at a laboratory energy of 50 MeV, shown in blue. The error bars represent method and model (EFT) uncertainties. The green band indicates the experimental phase shift^78^, while the purple line (band) indicate the mean result (one-sigma error) of the PREX experiment^5^. The dashed line indicates the linear trend of the ab initio points with *r*^2^ the coefficient of determination. **b** Correlation of neutron skin *R*_skin_( ^208^Pb) vs slope of the symmetry energy *L*. Relativistic and non-relativistic mean-field calculations are indicated with open symbols^79^, while ab initio results using the 34 non-implausible samples are indicated with filled circles. Experimental extractions of *R*_skin_( ^208^Pb) shown in the figure are from PREX^5^, MAMI^4^, RCNP^34^, $\bar{p}$^35^, and GW17081736^36^. All of these results involve modeling input as the neutron skin thickness cannot be measured directly. The quoted experimental error bars include statistical and some systematical uncertainties except for Ref. ^35^ that is statistical only and the GW170817 constraint which is a 90% upper bound from relativistic mean-field modeling of the tidal polarizability extracted in Ref. ^36^.

We also make predictions for nuclear-matter properties by employing the CC method on a momentum space lattice^37^ with a Bayesian machine-learning error model to quantify the uncertainties from the EFT truncation^14^ and the CC method (see Methods and Extended Data Fig. 6 for details). The observables we compute are the saturation density *ρ*_0_, the energy per nucleon of symmetric nuclear matter *E*_0_/*A*, its compressibility *K*, the symmetry energy *S* (that is, the difference between the energy per nucleon of neutron matter and symmetric nuclear matter), and its slope *L*. The posterior predictive distributions for these observables are shown in Fig. 3a. These distributions include samples from the relevant method and model error terms. Overall, we reveal relevant correlations among observables, previously indicated in mean-field models, and find good agreement with empirical bounds^38^. The last row shows the resulting correlations with *R*_skin_(^208^Pb) in our ab initio framework. In particular, we find essentially the same correlation between *R*_skin_(^208^Pb) and *L* as observed in mean-field models (Extended Data Fig. 7b).

## Discussion

**Extended Data Fig. 8:**
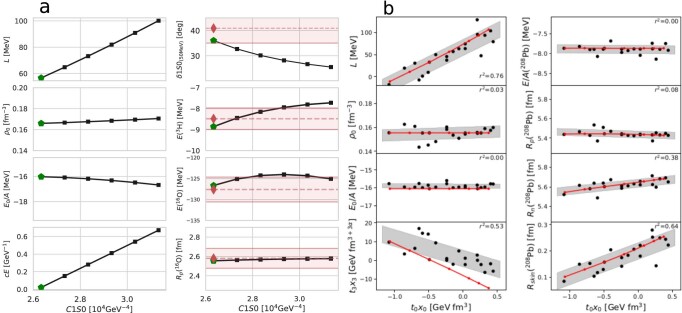
Parameter sensitivities in ab initio models and Skyrme parametrizations. **a**, Tuning the *C*_1*S*0_ LEC in our ab initio model to adjust the symmetry energy slope parameter *L* while compensating with the three-nucleon contact *c*_*E*_ to maintain the saturation density *ρ*_0_ and energy per nucleon *E*_0_/*A* of symmetric nuclear matter. The green pentagons correspond to results with one of the 34 interaction samples while the black squares indicate the results after tuning the *C*_1*S*0_ and *c*_*E*_ of that interaction. The right column shows the scattering phase shift *δ* in the ^1^*S*_0_ channel at 50 MeV, the ground-state energies in ^3^H and ^16^O and the point-proton radius *R*_p_ in ^16^O. The red diamonds and the dashed lines indicate the experimental values of target observables and the red bands indicate the corresponding *c*_*I*_ = 3 non-implausible regions, see Eq. (4), Extended Data Table 1 and Extended Data Figure 2. **b**, Illustration of the freedom in Skyrme parametrizations to adjust *L* while preserving *ρ*_0_ and *E*_0_/*A*. The parameters *x*_0_, *t*_0_, *x*_3_, *t*_3_ correspond to the functional form given in e.g.^80^. The black circles correspond to different parameter sets, while the red line indicates the result of starting with the SKX interaction and modifying the *x*_0_, *x*_3_ parameters while maintaining the binding energy per nucleon *E*/*A* of ^208^Pb. The right column also shows the ^208^Pb point-proton and point-neutron radii (*R*_p_ and *R*_n_, respectively) and neutron skin thickness *R*_skin_ for different parametrisations. The gray bands indicate a linear fit to the black points with *r*^2^ the coefficient of determination. Skyrme parameter sets included are SKX, SKXCSB^9^, SKI, SKII^81^, SKIII-VI^82^, SKa, SKb^83^, SKI2, SKI5^84^, SKT4, SKT6^85^, SKP^86^, SGI, SGII^87^, MSKA^88^, SKO^89^, SKM^*^^90^.

The predicted range of the ^208^Pb neutron skin thickness (Extended Data Table 2) is consistent with several extractions^4,39,40^, each of which involves some model dependence, and in mild tension (approximately 1.5*σ*) with the recent PREX result^5^. Ab initio computations yield a thin skin and a narrow range because the isovector physics is constrained by scattering data^8,13,41^. A thin skin was also predicted in ^48^Ca (ref. ^15^). We find that both *R*_skin_(^208^Pb) = 0.14–0.20 fm and the slope parameter *L* = 38–69 MeV are strongly correlated with scattering in the ^1^*S*_0_ partial wave for laboratory energies around 50 MeV (the strongest two-neutron channel allowed by the Pauli principle, with the energy naively corresponding to the Fermi energy of neutron matter at 0.8*ρ*_0_) (Extended Data Fig. 7a). It is possible, analogous to findings in mean-field theory^1,42^, to increase *L* beyond the range predicted in this work by tuning a contact in the ^1^*S*_0_ partial wave and simultaneously readjusting the three-body contact to maintain realistic nuclear saturation. However, this large slope *L* and increased *R*_skin_ come at the cost of degraded ^1^*S*_0_ scattering phase shifts, well beyond the corrections expected from higher-order terms (Extended Data Fig. 8). The large range of *L* and *R*_skin_ obtained in mean-field theory is a consequence of scattering data not being incorporated. It will be important to confront our predictions with more precise experimental measurements^43,44^. If the tension between scattering data and neutron skins persists, it will represent a serious challenge to our ab initio description of nuclear physics.

Our work demonstrates that ab initio approaches using nuclear forces from chiral EFT can consistently describe data from nucleon–nucleon scattering, few-body systems and heavy nuclei within the estimated theoretical uncertainties. Information contained in nucleon–nucleon scattering significantly constrains the properties of neutron matter. This same information constrains neutron skins, which provide a non-trivial empirical check on the reliability of ab initio predictions for the neutron-matter equation of state. Moving forward, it will be important to extend these calculations to higher orders in the EFT, both to further validate the error model and to improve precision, and to push the cut-off to higher values to confirm regulator independence. The framework presented herein will enable predictions with quantified uncertainties across the nuclear chart, advancing towards the goal of a single unified framework for describing low-energy nuclear physics.

## Methods

### Hamiltonian and model space

The many-body approaches used in this work, viz. CC, IMSRG, and many-body perturbation theory (MBPT), start from the intrinsic Hamiltonian

2 $$H={T}_{{{{\rm{kin}}}}}-{T}_{{{{\rm{CoM}}}}}+{V}_{{{{\rm{NN}}}}}+{V}_{{{{\rm{3N}}}}},$$

where *T*_kin_ is the kinetic energy, *T*_CoM_ is the kinetic energy of the centre of mass, *V*_NN_ is the nucleon–nucleon interaction and *V*_3N_ is the 3N interaction. To facilitate the convergence of heavy nuclei, the interactions employed in this work used a non-local regulator with a cut-off *Λ* = 394 MeV *c*^−1^. Specifically, the *V*_NN_ regulator is $f(p)=\exp {({p}^{2}/{{{\varLambda }}}^{2})}^{n}$ and the *V*_3N_ regulator is $f(p,q)=\exp {[-({p}^{2}+3{q}^{2}/4)/{{{\varLambda }}}^{2}]}^{n}$ with *n* = 4. Results should be independent of this choice, up to higher-order corrections, provided renormalization group invariance of the EFT. However, increasing the momentum scale of the cut-off leads to harder interactions, considerably increasing the required computational effort. We represent the 34 non-implausible interactions that resulted from the history-matching analysis in the Hartree–Fock basis in a model space of up to 15 major harmonic oscillator shells ($e=2n+l\le {e}_{\max }=14$, where *n* and *l* denote the radial and orbital angular momentum quantum numbers, respectively) with oscillator frequency *ℏω* = 10 MeV. Due to storage limitations, the 3N force had an additional energy cut given by ${e}_{1}+{e}_{2}+{e}_{3}\le {E}_{3\max }=28$. After obtaining the Hartree–Fock basis for each of the 34 non-implausible interactions, we capture 3N force effects via the normal-ordered two-body approximation before proceeding with the CC, IMSRG and MBPT calculations^46,47^. The convergence behaviour in ${e}_{\max }$ and ${E}_{{{{\rm{3max}}}}}$ is illustrated in Extended Data Fig. 3, where we use an interaction with a high likelihood that generates a large correlation energy. Thus, its convergence behaviour represents the worst case among the 34 non-implausible interactions. The model space converged results are investigated with ${E}_{{{{\rm{3max}}}}}\to 3{e}_{\max }$ and ${e}_{\max }\to \infty$ extrapolations. The functions ${E}_{{{{\rm{gs}}}}}\approx {c}_{0}{e}^{-{[({E}_{{{{\rm{3max}}}}}-{c}_{1})/{c}_{2}]}^{2}}+{E}_{{{{\rm{gs}}}}}({E}_{{{{\rm{3max}}}}}=\infty )$ and ${E}_{{{{\rm{gs}}}}}\approx {d}_{0}{e}^{-{d}_{1}{L}_{{{{\rm{IR}}}}}}+{E}_{{{{\rm{gs}}}}}({e}_{\max }=\infty )$ with ${L}_{{{{\rm{IR}}}}}=\sqrt{2({e}_{\max }+7/2)b}$ (where *b* is the harmonic oscillator length and the *c*_*i*_ and *d*_*i*_ are the fitting parameters) are used as the asymptotic forms for ${E}_{{{{\rm{3max}}}}}$ (ref. ^19^) and ${e}_{\max }$ ^48,49^), respectively. Through the extrapolations, the ground-state energies computed with ${e}_{\max }=14$ and ${E}_{{{{\rm{3max}}}}}=28$ are shifted by −75 ± 60 MeV. Likewise, the extrapolations of proton and neutron radii with the functional form given in refs. ^19^^,^^48,49^ yield a small (+0.005 ± 0.010 fm) shift of the neutron skin thickness.

### IMSRG calculations

The IMSRG calculations^32^^,^^50^ were performed at the IMSRG(2) level, using the Magnus formulation^51^. Operators for the point-proton and point-neutron radii, form factors and the electric dipole operator were consistently transformed. The dipole polarizablility *α*_D_ was computed using the equations-of-motion (EOM) method truncated at the two-particle-two-hole level (that is, the EOM-IMSRG(2,2) approximation^52^) and the Lanczos continued fraction method^53^. We compute the weak and charge form factors using the parameterization presented in ref. ^54^, though the form given in ref. ^55^ yields nearly identical results.

### MBPT calculations

MBPT theory calculations for ^208^Pb were performed in the Hartree–Fock basis to third order for the energies and to second order for radii.

### CC calculations

The CC calculations of ^208^Pb were truncated at the singles-and-doubles excitation level, known as the CCSD approximation^12,30,31^. We estimated the contribution from triples excitations to the ground-state energy of ^208^Pb as 10% of the CCSD correlation energy (which is a reliable estimate for closed-shell systems^31^).

Extended Data Fig. 4 compares the different many-body approaches used in this work (that is CC, IMSRG and MBPT) and allows us to estimate the uncertainties related to our many-body approach in computing the ground-state observables for ^208^Pb. The point proton and neutron radii are computed as ground-state expectation values (see, for example, ref. ^15^ for details). For ^48^Ca, we used a Hartree–Fock basis consisting of 15 major oscillator shells with an oscillator spacing of *ℏω* = 16 MeV, while for 3N forces we used ${E}_{{{{\rm{3max}}}}}=16$, which is sufficiently large to obtain converged results in this mass region. Here, we computed the ground-state energy using the Λ-CCSD(T) approximation^56^, which include perturbative triples corrections. The 2^+^ excited state in ^48^Ca was computed using the EOM CCSD approach^57^, and we estimated a −1 MeV shift from triples excitations based on EOM-CCSD(T) calculations of ^48^Ca and ^78^Ni using similar interactions^58^.

For the history-matching analysis, we used an emulator for the ^16^O ground-state energy and charge radius that was constructed using the recently developed sub-space projected (SP) CC method^29^. For higher precision in the emulator, we went beyond the SP-CCSD approximation used in ref. ^29^ and included leading-order triples excitations via the CCSDT-3 method^59^. The CCSDT-3 ground-state training vectors for ^16^O were obtained starting from the Hartree–Fock basis of the recently developed chiral interaction ΔNNLO_GO_(394) of ref. ^60^ in a model space consisting of 11 major harmonic oscillator shells with oscillator frequency *ℏω* = 16 MeV and ${E}_{{{{\rm{3max}}}}}=14$. The emulator used in the history matching was constructed by selecting 68 different training points in the 17-dimensional space of LECs using a space-filling Latin hypercube design with a 10% variation around the ΔNNLO_GO_(394) LECs. At each training point, we then performed a CCSDT-3 calculation to obtain the training vectors, for which we then construct the sub-space projected norm and Hamiltonian matrices. Once the SP-CCSDT-3 matrices are constructed, we may obtain the ground-state energy and charge radii for any target values of the LECs by diagonalizing a 68 × 68 generalized eigenvalue problem (see ref. ^29^ for more details). We checked the accuracy of the emulator by cross-validation against full-space CCSDT-3 calculations as demonstrated in Extended Data Fig. 4a and found a relative error that was smaller than 0.2%.

The nuclear-matter equation of state and saturation properties are computed with the CCD(T) approximation which includes doubles excitations and perturbative triples corrections. The 3N forces are considered beyond the normal-ordered two-body approximation by including the residual 3N force contribution in the triples. The calculations are performed on a cubic lattice in momentum space with periodic boundary conditions. The model space is constructed with ${(2{n}_{\max }+1)}^{3}$ momentum points, and we use ${n}_{\max }=4(3)$ for pure neutron matter (symmetric nuclear matter) and obtain converged results. We perform calculations for systems of 66 neutrons (132 nucleons) for pure neutron matter (symmetric nuclear matter) since results obtained with those particle numbers exhibit small finite-size effects^37^.

### Iterative history matching

In this work, we use an iterative approach known as history matching^25,26^ in which the model, solved at different fidelities, is confronted with experimental data *z* using equation (1). Obviously, we do not know the exact values of the errors in equation (1), hence we represent them as random variables and specify reasonable forms for their statistical distributions, in alignment with the Bayesian paradigm.

For many-body systems, we employ quantified method and (*A* = 16) emulator errors, as discussed above and summarized in Extended Data Table 1. For *A* ≤ 4 nuclei, we use the no-core shell model in Jacobi coordinates^61^ and eigenvector continuation emulators^28^. The associated method and emulator errors are very small. Probabilistic attributes of the model discrepancy terms are assigned based on the expected EFT convergence pattern^62,63^. For the history-matching observables considered here, we use point estimates of model errors from ref. ^64^.

The aim of history matching is to estimate the set ${{{\mathcal{Q}}}}(z)$ of parameterizations *θ* for which the evaluation of a model *M*(*θ*) yields an acceptable (or at least not implausible) match to a set of observations *z*. History matching has been employed in various studies involving complex computer models^65–68^ ranging, for example, from the effects of climate modelling^69,70^ to systems biology^71^.

We introduce the individual implausibility measure

3 $${I}_{i}^{2}(\theta )=\frac{| {M}_{i}(\theta )-{z}_{i}{| }^{2}}{{{{\rm{Var}}}}\left({M}_{i}(\theta )-{z}_{i}\right)},$$

which is a function over the input parameter space and quantifies the (mis-)match between our (emulated) model output *M*_*i*_(*θ*) and the observation *z*_*i*_ for an observable in the target set ${{{\mathcal{Z}}}}$. We mainly employ a maximum implausibility measure as the restricting quantity. Specifically, we consider a particular value for *θ* as implausible if

4 $${I}_{M}(\theta )\equiv \mathop{\max }\limits_{{z}_{i}\in {{{\mathcal{Z}}}}}{I}_{i}(\theta ) > {c}_{I},$$

with *c*_*I*_ ≡ 3.0, appealing to Pukelheim’s three-sigma rule^72^. In accordance with the assumptions leading to equation (1), the variance in the denominator of equation (3) is a sum of independent squared errors. Generalizations of these assumptions are straightforward if additional information on error covariances or possible inaccuracies in our error model would become available.

An important strength of the history matching is that we can proceed iteratively, excluding regions of input space by imposing cut-offs on implausibility measures that can include additional observables *z*_*i*_ and corresponding model outputs *M*_*i*_ with possibly refined emulators as the parameter volume is reduced. The history-matching process is designed to be independent of the order in which observables are included, as is discussed in ref. ^67^. This is an important feature because it allows for efficient choices regarding such orderings. The iterative history matching proceeds in waves according to a straightforward strategy that can be summarized as follows:

1. At wave *j*: Evaluate a set of model runs over the current non-implausible (NI) volume ${{{{\mathcal{Q}}}}}_{j}$ using a space-filling design of sample values for the parameter inputs *θ*. Choose a rejection strategy based on implausibility measures for a set ${{{{\mathcal{Z}}}}}_{j}$ of informative observables.
2. Construct or refine emulators for the model predictions across ${{{{\mathcal{Q}}}}}_{j}$.
3. The implausibility measures are then calculated over ${{{{\mathcal{Q}}}}}_{j}$ using the emulators, and implausibility cut-offs are imposed. This defines a new, smaller non-implausible volume ${{{{\mathcal{Q}}}}}_{j+1}$ which should satisfy ${\mathcal{Q}}_{j+1}\subset {\mathcal{Q}}_{j}$.
4. Unless (a) computational resources are exhausted or (b) all considered points in the parameter space are deemed implausible, we may include additional informative observables in the considered set ${{{{\mathcal{Z}}}}}_{j+1}$, and return to step 1.
5. If step 4(a) is true, we generate a number of acceptable runs from the final non-implausible volume ${{{{\mathcal{Q}}}}}_{{{{\rm{final}}}}}$, sampled according to scientific need.

The ab initio model for the observables we consider includes at most 17 parameters: 4 subleading pion–nucleon couplings, 11 nucleon–nucleon contact couplings and two short-ranged 3N couplings. To identify a set of non-implausible parameter samples, we performed iterative history matching in four waves using observables and implausibility measures, as summarized in Extended Data Fig. 1b. For each wave, we employ a sufficiently dense Latin hypercube set of several million candidate parameter samples. For the model evaluations, we utilized fast computations of neutron–proton scattering phase shifts and efficient emulators for the few- and many-body history-matching observables. See Extended Data Table 1 and Extended Data Fig. 2 for the list of history-matching observables and information on the errors that enter the implausibility measure in equation (3). The input volume for wave 1 incorporates the naturalness expectation for LECs, but still includes large ranges for the relevant parameters as indicated by the panel ranges in Extended Data Fig. 1a. In all four waves, the input volume for *c*_1,2,3,4_ is a four-dimensional hypercube mapped onto the multivariate Gaussian probability density function (PDF) resulting from a Roy–Steiner analysis of pion–nucleon scattering data^73^. In wave 1 and wave 2, we sampled all relevant parameter directions for the set of included two-nucleon observables. In wave 3, the ^3^H and ^4^He observables were added such that the 3N force parameters *c*_D_ and *c*_E_ can also be constrained. Since these observables are known to be rather insensitive to the four model parameters acting solely in *P* waves, we ignored this subset of the inputs and compensated by slightly enlarging the corresponding method errors. This is a well-known emulation procedure called inactive parameter identification^25^. For wave 4, we considered all 17 model parameters and added the ground-state energy and radius of ^16^O to the set ${{{{\mathcal{Z}}}}}_{4}$ and emulated the model outputs for 5 × 10^8^ parameter samples. By including oxygen data, we explore the modelling capabilities of our ab initio approach. Extended Data Fig. 1a summarizes the sequential non-implausible volume reduction, wave-by-wave, and indicates the set of 4,337 non-implausible samples after the fourth wave. Note that the use of history matching would, in principle, allow a detailed study of the information content of various observables in heavy-mass nuclei. Such an analysis, however, requires an extensive set of reliable emulators and lies beyond the scope of the present work. The volume reduction is determined by the maximum implausibility cut-off in equation (4) with additional confirmation from the optical depths (which indicate the density of non-implausible samples; see equations (25) and (26) in ref. ^71^). The non-implausible samples summarize the parameter region of interest and can directly provide insight regarding the interdependences between parameters induced by the match to observed data. This region is also where we would expect the posterior distribution to reside, and we note that our history-matching procedure has allowed us to reduce its size by more than seven orders of magnitude compared with the prior volume (Extended Data Fig. 1b).

As a final step, we confront the set of non-implausible samples from wave 4 with neutron–proton scattering phase shifts such that our final set of non-implausible samples has been matched with all history-matching observables. For this final implausibility check, we employ a slightly less strict cut-off and allow the first, second and third maxima of *I*_*i*_(*θ*) (for ${z}_{i}\in {{{{\mathcal{Z}}}}}_{{{{\rm{final}}}}}$) to be 5.0, 4.0 and 3.0, respectively, accommodating the more extreme maxima we may anticipate when considering a significantly larger number of observables. The end result is a set of 34 non-implausible samples that we use for predicting ^48^Ca and ^208^Pb observables, as well as the equation of state of both symmetric nuclear matter and pure neutron matter.

### Posterior predictive distributions

The 34 non-implausible samples from the final history-matching wave are used to compute energies, radii of proton and neutron distributions and electric dipole polarizabilities (*α*_D_) for ^48^Ca and ^208^Pb. They are also used to compute the electric and weak charge form factors for the same nuclei at a relevant momentum transfer, and the energy per particle of infinite nuclear matter at various densities to extract key properties of the nuclear equation of state (see below). These results are shown in Fig. 3(blue circles).

To make quantitative predictions, with a statistical interpretation, for *R*_skin_(^208^Pb) and other observables, we use the same 34 parameter sets to extract representative samples from the posterior PDF $p(\theta | {{{{\mathcal{D}}}}}_{{{{\rm{cal}}}}})$. Bulk properties (energies and charge radii) of ^48^Ca together with the structure-sensitive 2^+^ excited-state energy of ^48^Ca are used to define the calibration data set ${{{{\mathcal{D}}}}}_{{{{\rm{cal}}}}}$. The IMSRG and CC convergence studies make it possible to quantify the method errors. These are summarized in Extended Data Table 1. The EFT truncation errors are quantified by adopting the EFT convergence model^74,75^ for observable *y*

5 $$y={y}_{{{{\rm{ref}}}}}\left(\mathop{\sum }\limits_{i=0}^{k}{c}_{i}{Q}^{i}+\mathop{\sum }\limits_{i=k+1}^{\infty }{c}_{i}{Q}^{i}\right),$$

with observable coefficients *c*_*i*_ that are expected to be of natural size, and the expansion parameter *Q* = 0.42 following our Bayesian error model for nuclear matter at the relevant density (see below). The first sum in the parenthesis is the model prediction *y*_*k*_(*θ*) of observable *y* at truncation order *k* in the chiral expansion. The second sum than represents the model error because it includes the terms that are not explicitly included. We can quantify the magnitude of these terms by learning about the distribution for *c*_*i*_, which we assume to be described by a single normal distribution per observable type with zero mean and a variance parameter ${\bar{c}}^{2}$. We employ the nuclear-matter error analysis for the energy per particle of symmetric nuclear matter (described below) to provide the model error for *E*/*A* in ^48^Ca and ^208^Pb. For radii and electric dipole polarizabilities, we employ the next-to leading order and next-to-next-to leading order interactions of ref. ^60^ and compute these observables at both orders for various Ca, Ni and Sn isotopes. The reference values *y*_ref_ are set to *r*_0_ *A*^1/3^ for radii (with *r*_*0*_ = 1.2 fm) and to the experimental value for *α*_D_. From these data, we extract ${\bar{c}}^{2}$ and perform the geometric sum of the second term in equation (5). The resulting standard deviations for model errors are summarized in Extended Data Table 1.

At this stage, we can approximately extract samples from the parameter posterior $p(\theta | {{{{\mathcal{D}}}}}_{{{{\rm{cal}}}}})$ by employing the established method of sampling/importance resampling^33^^,^^76^. We assume a uniform prior probability for the non-implausible samples, and we introduce a normally distributed likelihood ${{{\mathcal{L}}}}({{{{\mathcal{D}}}}}_{{{{\rm{cal}}}}}| \theta )$, assuming independent experimental, method and model errors. The prior for *c*_1,2,3,4_ is the multivariate Gaussian resulting from a Roy–Steiner analysis of pion–nucleon scattering data^73^. Defining importance weights

6 $${q}_{i}={{{\mathcal{L}}}}({{{{\mathcal{D}}}}}_{{{{\rm{cal}}}}}| {\theta }_{i})/\mathop{\sum }\limits_{j=1}^{n}{{{\mathcal{L}}}}({{{{\mathcal{D}}}}}_{{{{\rm{cal}}}}}| {\theta }_{j}),$$

we draw samples θ^*^ from the discrete distribution {*θ*_1_, …, *θ*_*n*_} with probability mass *q*_*i*_ on *θ*_*i*_. These samples are then approximately distributed according to the parameter posterior that we are seeking^33^^,^^76^.

Although we are operating with a finite number of *n* = 34 representative samples from the parameter PDF, it is reassuring that about half of them are within a factor of two from the most probable one in terms of the importance weight (Extended Data Fig. 5). Consequently, our final predictions will not be dominated by a very small number of interactions. In addition, as we do not anticipate the parameter PDF to be of a particularly complex shape, based on the results of the history match, consideration of the various error structures in the analysis and on the posterior predictive distributions (PPDs) shown in Fig. 3, and as we are mainly interested in examining such lower one- or two-dimensional PPDs, this sample size was deemed sufficient and the corresponding sampling error assumed subdominant. We use these samples to draw corresponding samples from

7 $${{{{\rm{PPD}}}}}_{{{{\rm{parametric}}}}}=\{{y}_{k}(\theta ):\theta \sim p(\theta | {{{{\mathcal{D}}}}}_{{{{\rm{cal}}}}})\}.$$

This PPD is the set of all model predictions computed over likely values of the parameters, that is, drawing from the posterior PDF for *θ*. The full PPD is then defined, in analogy with equation (7), as the set evaluation of *y* which is the sum

8 $$y={y}_{k}+{\epsilon }_{{{{\rm{method}}}}}+{\epsilon }_{{{{\rm{model}}}}},$$

where we assume method and model errors to be independent of the parameters. In practice, we produce 10^4^ samples from this full PPD for *y* by resampling the 34 samples of the model PPD in equation (7) according to their importance weights, and adding samples from the error terms in equation (8). We perform model checking by comparing this final PPD with the data used in the iterative history-matching step, and in the likelihood calibration. In addition, we find that our predictions for the measured electric dipole polarizabilities of ^48^Ca and ^208^Pb as well as bulk properties of ^208^Pb serve as a validation of the reliability of our analysis and assigned errors (Fig. 2 and Extended Data Table 1).

In addition, we explored the sensitivity of our results to modifications of the likelihood definition. Specifically, we used a Student *t* distribution (*ν* = 5) to see the effects of allowing heavier tails, and we introduced an error covariance matrix to study the effect of possible correlations (with *ρ* ≈ 0.7) between the errors in the binding energy and radius of ^48^Ca. In the end, the differences in the extracted credibility regions was ~1%, and we therefore present only results obtained with the uncorrelated, multivariate normal distribution.

Our final predictions for *R*_skin_(^208^Pb), *R*_skin_(^48^Ca) and nuclear-matter properties are presented in Fig. 3 and Extended Data Table 2. For these observables, we use the Bayesian machine learning error model described below to assign relevant correlations between equation-of-state observables. For the model errors in *R*_skin_(^208^Pb) and *L*, we use a correlation coefficient of *ρ* = 0.9 as motivated by the strong correlation between the observables computed with the 34 non-implausible samples. Note that *S*, *L* and *K* are computed at the specific saturation density of the corresponding non-implausible interaction.

### Bayesian machine learning error model

Similar to equation (1), the predicted nuclear matter observables can be written as

9 $$y={y}_{k}(\rho )+{\varepsilon }_{k}(\rho )+{\varepsilon }_{{{{\rm{method}}}}}(\rho ),$$

where *y*_*k*_(*ρ*) is the CC prediction using our EFT model truncated at order *k*, *ε*_*k*_(*ρ*) is the EFT truncation (model) error and *ε*_method_(*ρ*) is the CC method error. In this work, we apply a Bayesian machine learning error model^14^ to quantify the density dependence of both the method and truncation errors. The error model is based on multi-task Gaussian processes that learn both the size and the correlations of the target errors from given prior information. Following a physically motivated Gaussian process (GP) model^14^, the EFT truncation errors *ε*_*k*_ at given density *ρ* are distributed as

10 $${\varepsilon }_{k}(\rho )\,| \,{\bar{c}}^{2},l,Q \sim {{{\rm{GP}}}}[0,{\bar{c}}^{2}{R}_{{\varepsilon }_{k}}(\rho ,\rho ^{\prime} ;l)],$$

with

11 $${R}_{{\varepsilon }_{k}}(\rho ,\rho ^{\prime} ;l)={y}_{{{{\rm{ref}}}}}(\rho ){y}_{{{{\rm{ref}}}}}(\rho ^{\prime} )\frac{{[Q(\rho )Q(\rho ^{\prime} )]}^{k+1}}{1-Q(\rho )Q(\rho ^{\prime} )}r(\rho ,\rho ^{\prime} ;l).$$

Here, *k* = 3 for the ΔNNLO(394) EFT model used in this work, while ${\bar{c}}^{2}$, *l* and *Q* are hyperparameters corresponding to the variance, the correlation length and the expansion parameter. Finally, we choose the reference scale *y*_ref_ to be the EFT leading-order prediction. The mean of the Gaussian process is set to be zero since the order-by-order truncation error can be either positive or negative and the correlation function $r(\rho ,\rho ^{\prime} ;l)$ in equation (11) is the Gaussian radial basis function.

We employ Bayesian inference to optimize the Gaussian process hyperparameters using order-by-order predictions of the equation of state for both pure neutron matter and symmetric nuclear matter with the Δ-full interactions from ref. ^64^. In this work, we find ${\bar{c}}_{{{{\rm{PNM}}}}}=0.99$ and *l*_PNM_ = 88 fm^−1^ for pure neutron matter and ${\bar{c}}_{{{{\rm{SNM}}}}}=1.66$ and *l*_SNM_ = 0.45 fm^−1^ for symmetric nuclear matter. This leads to *Q* = 0.41 when estimating the model errors for *E* / *A* in ^48^Ca and ^208^Pb.

The above Gaussian processes only describe the correlated structure of truncation errors for one type of nucleonic matter. In addition, the correlation between pure neutron matter and symmetric nuclear matter is crucial for correctly assigning errors to observables that involve both *E*/*N* and *E*/*A* (such as the symmetry energy *S*). For this purpose, we use a multitask Gaussian process that simultaneously describes the truncation errors of pure neutron matter and symmetric nuclear matter according to

12 $$\left[\begin{array}{c}{\varepsilon }_{k,{{{\rm{PNM}}}}}\\ {\varepsilon }_{k,{{{\rm{SNM}}}}}\end{array}\right] \sim {{{\rm{GP}}}}\left(\left[\begin{array}{c}0\\ 0\end{array}\right],\left[\begin{array}{cc}{K}_{11}&{K}_{12}\\ {K}_{21}&{K}_{22}\end{array}\right]\right),$$

where *K*_11_ and *K*_22_ are the covariance matrices generated from the kernel function ${\bar{c}}^{2}{R}_{{\varepsilon }_{k}}(\rho ,\rho ^{\prime} ;l)$ for pure neutron matter and symmetric nuclear matter, respectively, while *K*_12_ (*K*_21_) is the cross-covariance as in ref. ^77^.

Regarding the CC method error, different sources of uncertainty should be considered. The truncation error of the cluster operator (*ε*_cc_) and the finite-size effect (*ε*_fs_) are the main ones, and the total method error is then *ε*_method_ = *ε*_cc_ + *ε*_fs_. Following the Bayesian error model, we have the following general expression for the method error:

13 $${\varepsilon }_{{{{\rm{me}}}}}(\rho )\,| \,{\bar{c}}_{{{{\rm{me}}}}}^{2},{l}_{{{{\rm{me}}}}}, \sim {{{\rm{GP}}}}[0,{\bar{c}}_{{{{\rm{me}}}}}^{2}{R}_{{{{\rm{me}}}}}(\rho ,\rho ^{\prime} ;{l}_{{{{\rm{me}}}}})],$$

with

14 $${R}_{{{{\rm{me}}}}}(\rho ,\rho ^{\prime} ;{l}_{{{{\rm{me}}}}})={y}_{{{{\rm{me,ref}}}}}(\rho ){y}_{{{{\rm{me,ref}}}}}(\rho ^{\prime} )r(\rho ,\rho ^{\prime} ;{l}_{{{{\rm{me}}}}}).$$

Here, the subscript ‘me’ stands for either the cluster operator truncation ‘cc’ or the finite-size effect ‘fs’ method error. For the cluster operator truncation errors *ε*_cc_, the reference scale *y*_me,ref_ is taken to be the CCD(T) correlation energy. The Gaussian processes are then optimized with data from different interactions by assuming that the energy difference between CCD and CCD(T) can be used as an approximation of the cluster operator truncation error. The correlation lengths learned from the training data are *l*_me,PNM_ = 0.83 fm^−1^ for pure neutron matter and *l*_me,SNM_ = 0.39 fm^−1^ for symmetric nuclear matter. Based on the convergence study, we take ±10% of the correlation energy as the 95% credible interval, which gives ${\bar{c}}_{{{{\rm{me}}}}}=0.05$ for *ε*_cc_. For the finite-size effect *ε*_fs_, the reference scale is taken to be the CCD(T) ground-state energy. Then, following ref. ^37^, we use ±0.5% (±4%) of the ground-state energy of the pure neutron matter (the symmetric nuclear matter) as a conservative estimation of the finite-size effect (95% credible interval) when using periodic boundary conditions with 66 neutrons (132 nucleons) around the saturation point. This leads to ${\bar{c}}_{{{{\rm{me}}}},PNM}=0.0025$ and ${\bar{c}}_{{{{\rm{me}}}},SNM}=0.02$ for *ε*_fs_. The finite-size effects of different densities are clearly correlated, while there are insufficient data to learn its correlation structure. Here, we simply used 0.83 fm^−1^ (0.39 fm^−1^) as the correlation length for pure neutron matter (symmetric nuclear matter) and assume zero correlation between pure neutron matter and symmetric nuclear matter.

Once the model and method errors are determined, it is straightforward to sample these errors from the corresponding covariance matrix and produce the equation-of-state predictions using equation (9) for any given interaction. This sampling procedure is crucial for generating the posterior predictive distribution of nuclear-matter observables shown in Fig. 3a. The CCD(T) calculations for the nuclear-matter equation of state and the corresponding 2*σ* credible interval for the method and model errors are illustrated in Extended Data Fig. 6. The sampling procedure is made explicit with three randomly sampled equation-of-state predictions. Note that, even though the sampled errors for one given density appear to be random, the multi-task Gaussian processes will guarantee that the sampled equations of state of nuclear matter are smooth and properly correlated with each other.

## Online content

Any methods, additional references, Nature Research reporting summaries, source data, extended data, supplementary information, acknowledgements, peer review information; details of author contributions and competing interests; and statements of data and code availability are available at 10.1038/s41567-022-01715-8.

### Source data

Source Data Fig. 1 Year of computation and mass number divided by total TOP500 compute power.

Source Data Fig. 2 Finite nuclei predictions for the 34 non-implausible interactions.

Source Data Fig. 3 Infinite nuclear matter properties, *R*_skin_ and weak plus charge form factor predictions for the 34 non-implausible interactions (csv file). Mean vector and covariance matrix of a multivariate normal distribution that approximates the full posterior predictive distribution (txt file).

Source Data Extended Data Fig. 1 Parameters of the final 34 non-implausible interactions.

## Figures and Tables

**Extended Data Table 1:**
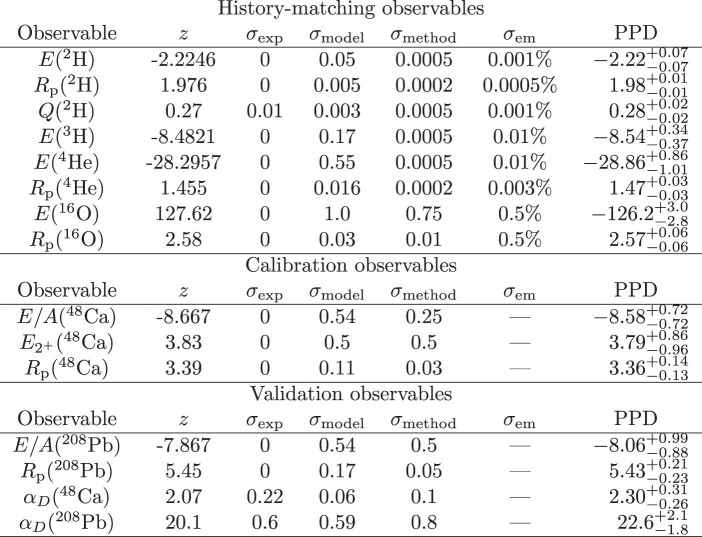
Error assignments, PPD model checking and validation. Experimental target values and error assignments for observables used in the fourth wave of the iterative history matching (history-matching observables), for the Gaussian likelihood calibration of the final non-implausible samples (calibration observables), and for model validation with predicted ^208^Pb observables and electric dipole polarizabilities (validation observables). Energies *E* (in MeV) with experimental targets from Refs. ^91,92^, point-proton radii *R*_p_ (in fm) with experimental targets translated from measured charge radii^93^ (see Ref. ^94^ for more details). For the deuteron quadrupole moment *Q* (in *e*^2^fm^2^) we use the theoretical result obtained from the high-precision meson-exchange nucleon-nucleon model CD-Bonn^95^ as a target with a 4% error bar. Electric dipole polarizability *α*_D_ (in fm^3^) with experimental targets from Refs. ^96,97^. Theoretical model (method) errors are estimated from the EFT (many-body) convergence pattern as discussed in the text. These theory errors have zero mean except for the excitation energy ${E}_{{2}^{+}}$(^48^Ca) with *μ*_method_ = − 1 MeV from estimated triples and *E*/*A*(^208^Pb) with *μ*_method_ = − 0.36 MeV/A from ${e}_{\max }$ model-space extrapolation. Emulator errors are estimated from cross validation. All errors are represented by the estimated standard deviation of the corresponding random vartiable: ${\sigma }_{\exp }={{{\rm{Var}}}}{\left[{\varepsilon }_{\exp }\right]}^{1/2}$, ${\sigma }_{{{{\rm{model}}}}}={{{\rm{Var}}}}{\left[{\varepsilon }_{{{{\rm{model}}}}}\right]}^{1/2}$, etc. Most of the experimental errors are negligible compared to the theoretical ones and therefore given as ${\sigma }_{\exp }=0$. We assume that all theory errors are parametrization independent. The final model predictions from the PPD described in the text (and shown in Fig. 2) are summarized by the medians and the marginal 68% credibility regions in the last column.

**Extended Data Table 2:**
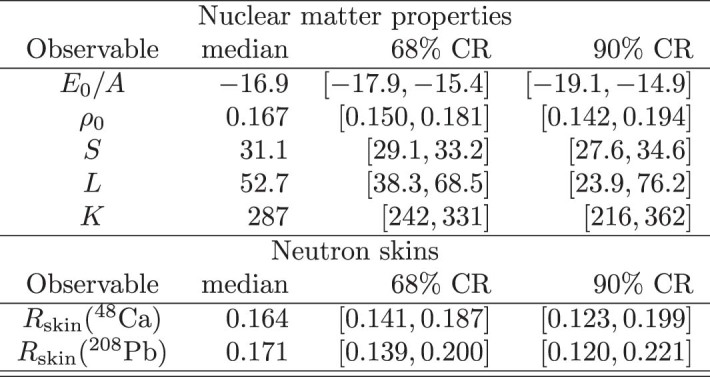
Predictions for the nuclear equation of state at saturation density and for neutron skins. Medians and 68%, 90% credible regions (CR) for the final PPD including samples from the error models (see also Fig. 3 and text for details). The saturation density, *ρ*_0_, is in (fm^−3^), the neutron skin thickness, *R*_skin_( ^208^Pb) and *R*_skin_( ^48^Ca), in (fm), while the saturation energy per particle (*E*_0_/*A*), the symmetry energy (*S*), its slope (*L*), and incompressibility (*K*) at saturation density are all in (MeV). Empirical regions shown in Fig. 3 are *E*_0_/*A* = − 16.0 ± 0.5, *ρ*_0_ = 0.16 ± 0.01, *S* = 31 ± 1, *L* = 50 ± 10 and *K* = 240 ± 20 from Refs. ^38,98,99^.

## Data Availability

Source data for Figs. 1, 2 and 3a–c are provided with this paper. Furthermore, the parameters of the 34 non-implausible interactions that is the final result of Extended Data Fig. 1 plus the mean vector and covariance matrix of a multivariate normal distribution that approximates the full posterior predictive distribution shown in Fig. 3a are also provided. The data that support the other figures of this study are available from the corresponding author upon reasonable request. Source data are provided with this paper.

## Code availability

The code used to perform the IMSRG calculations is available at https://github.com/ragnarstroberg/imsrg. Enquiries about other codes used in this work should be adressed to the corresponding author.
