# Author Correction: Ab initio predictions link the neutron skin of ^208^Pb to nuclear forces

**DOI:** 10.1038/s41567-023-02324-9

**Published:** 2023-11-20

**Authors:** Baishan Hu, Weiguang Jiang, Takayuki Miyagi, Zhonghao Sun, Andreas Ekström, Christian Forssén, Gaute Hagen, Jason D. Holt, Thomas Papenbrock, S. Ragnar Stroberg, Ian Vernon

**Affiliations:** 1 TRIUMF, Vancouver, British Columbia Canada; 2 Department of Physics, Chalmers University of Technology, Gothenburg, Sweden; 3 Department of Physics, Technische Universität Darmstadt, Darmstadt, Germany; 4 ExtreMe Matter Institute EMMI, GSI Helmholtzzentrum für Schwerionenforschung GmbH, Darmstadt, Germany; 5 Department of Physics and Astronomy, University of Tennessee, Knoxville, TN USA; 6 Physics Division, Oak Ridge National Laboratory, Oak Ridge, TN USA; 7 Department of Physics, McGill University, Montreal, Quebec Canada; 8 Department of Physics, University of Washington, Seattle, WA USA; 9 Physics Division, Argonne National Laboratory, Lemont, IL USA; 10 Department of Mathematical Sciences, Durham University, Durham, UK

**Keywords:** Theoretical nuclear physics, Astronomy and astrophysics

Correction to: *Nature Physics* 10.1038/s41567-022-01715-8, published online 22 August 2022.

The initially published version of the paper contained an error. Matrix elements in the normal-ordering procedure of the three-nucleon force were computed incorrectly, which influences results presented in Fig. 3a. The figure has been corrected, and the Source Data file for Fig. 3 has been replaced. These changes have no effect on the conclusions drawn in the article regarding the neutron skin thickness of ^208^Pb and other properties of finite nuclei.

The fourth sentence in the Discussion now starts “We find that both *R*_skin_ (^208^Pb) = 0.14–0.20 fm and the slope parameter *L* = 38–69 MeV are strongly correlated with scattering in the ^1^*S*_0_ partial wave for laboratory energies around 50 MeV”, replacing the original wording “We find that both *R*_skin_ (^208^Pb) = 0.14–0.20 fm and the slope parameter *L* = 37–66 MeV are strongly correlated with scattering in the ^1^*S*_0_ partial wave for laboratory energies around 50 MeV”.

The error also affects results presented in Methods, Extended Data Table 2 and Extended Data Figs. 6–8.

The final two sentences in the third paragraph of the “Bayesian machine learning error model” section (in Methods) now read “In this work, we find ${\bar{{\rm{c}}}}_{{PNM}}=0.99$ and *l*_*PNM*_ = 0.88 fm^−1^ for pure neutron matter and ${\bar{{\rm{c}}}}_{{SNM}}=1.66$ and ${l}_{{SNM}}=0.45$ fm^−1^ for symmetric nuclear matter. This leads to *Q* = 0.41 when estimating the model errors for *E*/*A* in ^48^Ca and ^208^Pb”, replacing the original wording “In this work, we find ${\bar{{\rm{c}}}}_{{PNM}}=1.00$ and ${l}_{{PNM}}=0.92$ fm^−1^ for pure neutron matter and ${\bar{{\rm{c}}}}_{{SNM}}=1.55$ and *l*_*SNM*_ = 0.48 fm^−1^ for symmetric nuclear matter.”

Furthermore, the fourth sentence after Eq. (14) that reads “The correlation lengths learned from the training data are *l*_*me*,*PNM*_ = 0.83 fm^−1^ for pure neutron matter and *l*_*me,SNM*_ = 0.39 fm^−1^ for symmetric nuclear matter.” was changed from “The correlation lengths learned from the training data are *l*_*me,PNM*_ = 0.81 fm^−1^ for pure neutron matter and *l*_*me,SNM*_ = 0.34 fm^−1^ for symmetric nuclear matter.”

Finally, the last sentence of the same paragraph now starts with “Here we simply used $0.83$ fm^−1^ (0.39 fm^−1^) as the correlation length …” which was changed from the original text “Here we simply used $0.81$ fm^−1^ (0.34 fm^−1^) as the correlation length …”.

All results that involve predictions for properties of infinite nuclear matter have been corrected. Predictions for properties of finite nuclei, including the thickness of the neutron skin, are not affected. The original and corrected versions of Fig. 3, Extended Data Table 2 and Extended Data Figs. 6, 7b and 8a are shown in the Supplementary Information for this amendment, and the errors have been corrected in the HTML and PDF versions of the article.

Supplementary Information is available in the online version of this amendment.

### Supplementary information

Supplementary information Original and revised Fig. 3, Extended Data Figs. 6, 7b, 8a and Extended Data Table 2

