# Supplementary material for: Author Correction: Ab initio predictions link the neutron skin of 208Pb to nuclear forces
Source: Nat Phys. 2023 Nov 20;20(1):169. doi: 10.1038/s41567-023-02324-9 (PMC10791583; doi:10.1038/s41567-023-02324-9)
Supplement: Supplementary file 1 — Original and revised Fig. 3, Extended Data Figs. 6, 7b, 8a and Extended Data Table 2 [file 41567_2023_2324_MOESM1_ESM.pdf]

Author Correction: Ab initio predictions link the neutron skin of ^{208}Pb to nuclear forces

In the format provided by the authors and unedited

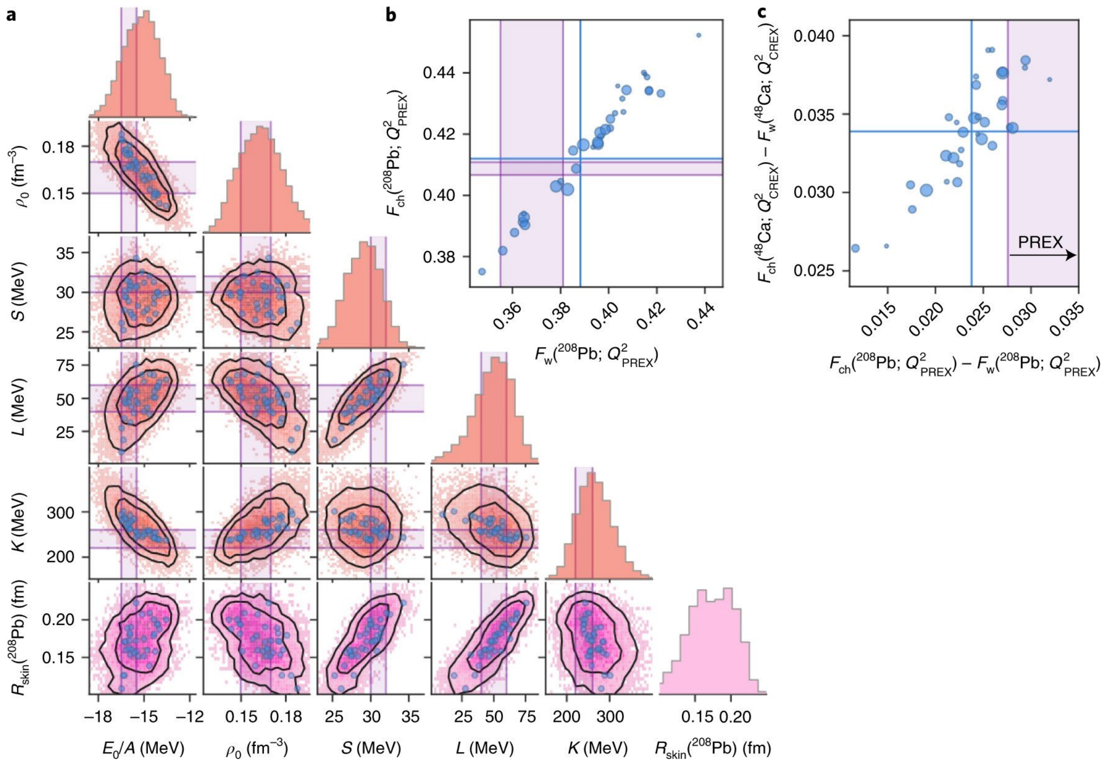

Original Fig. 3

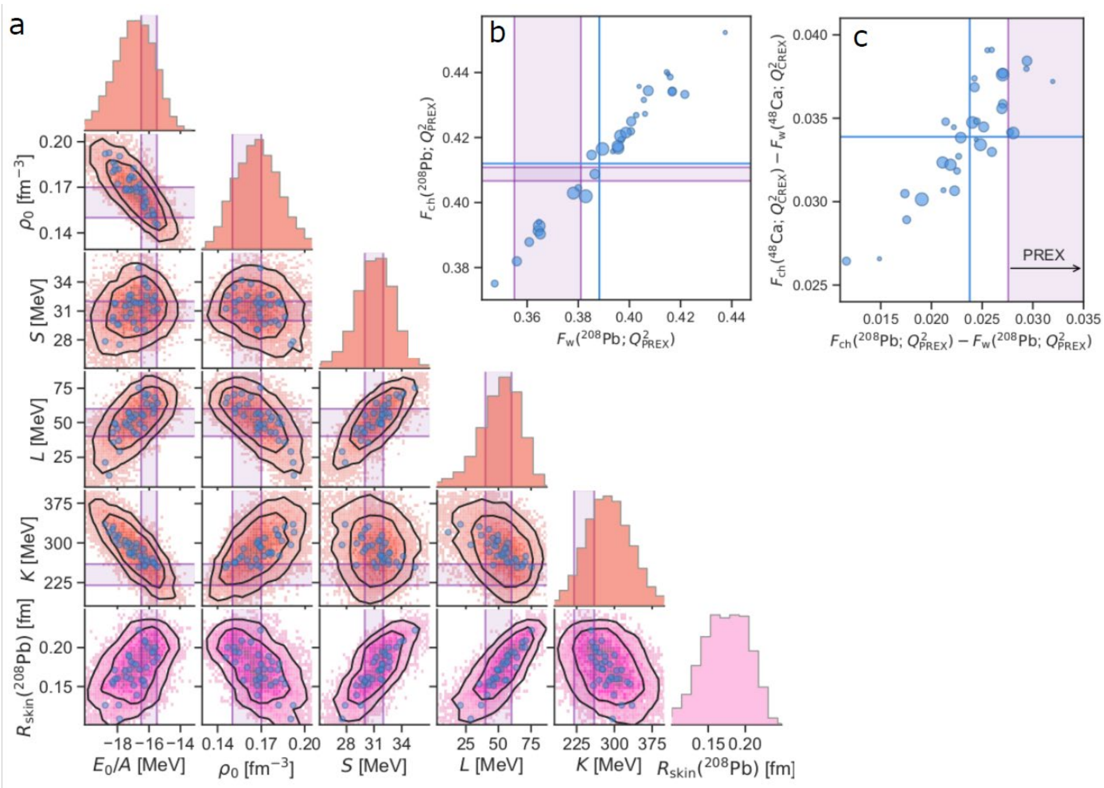

Revised Fig. 3

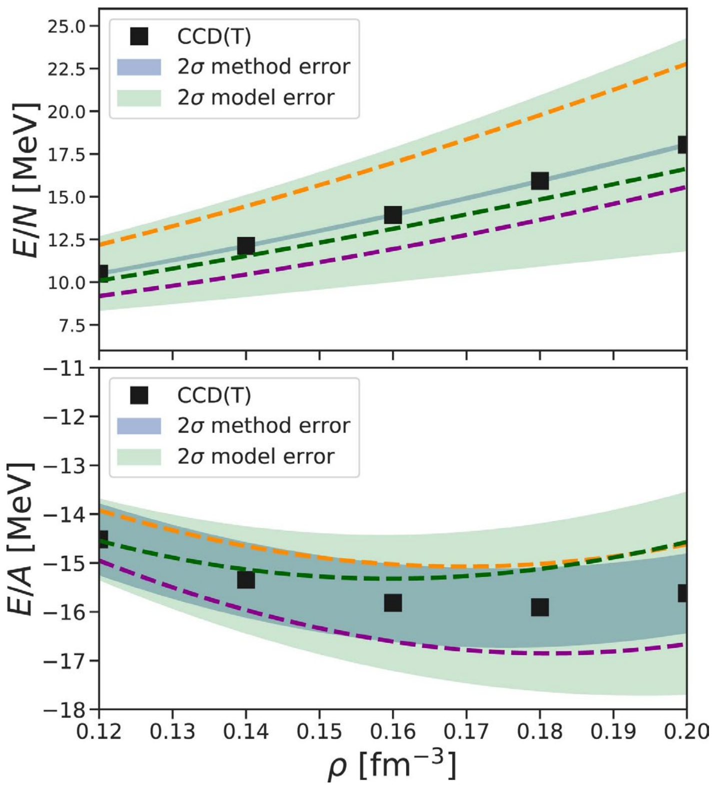

Original Extended Data Fig. 6

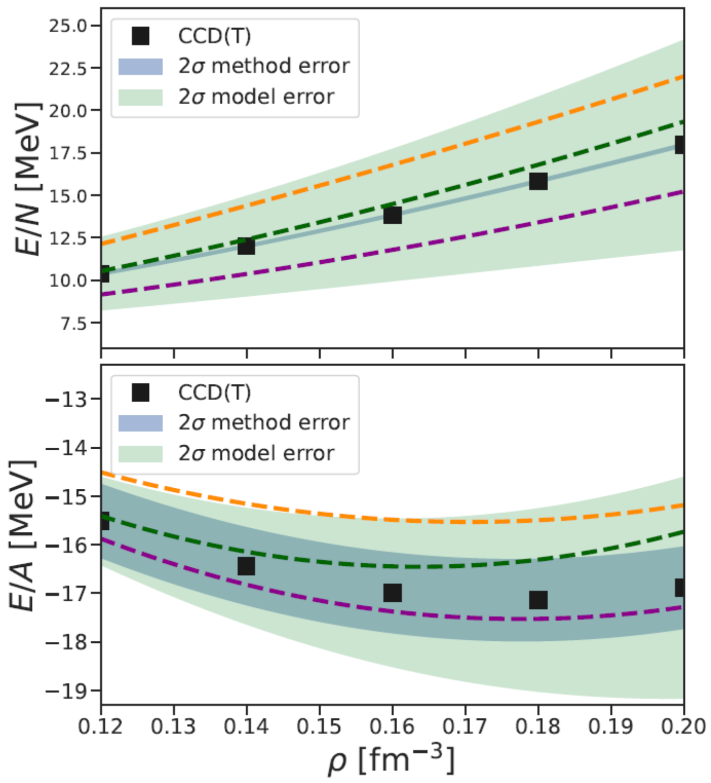

Revised Extended Data Fig. 6

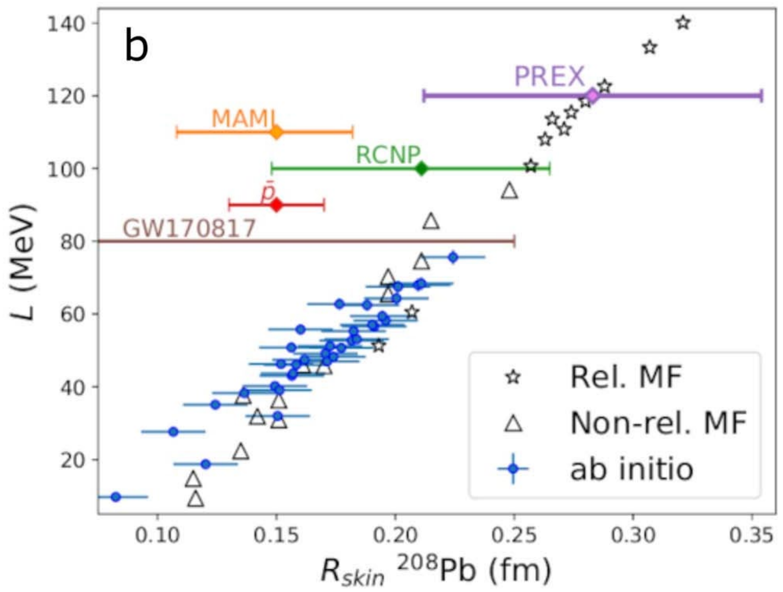

Original Extended Data Fig. 7b

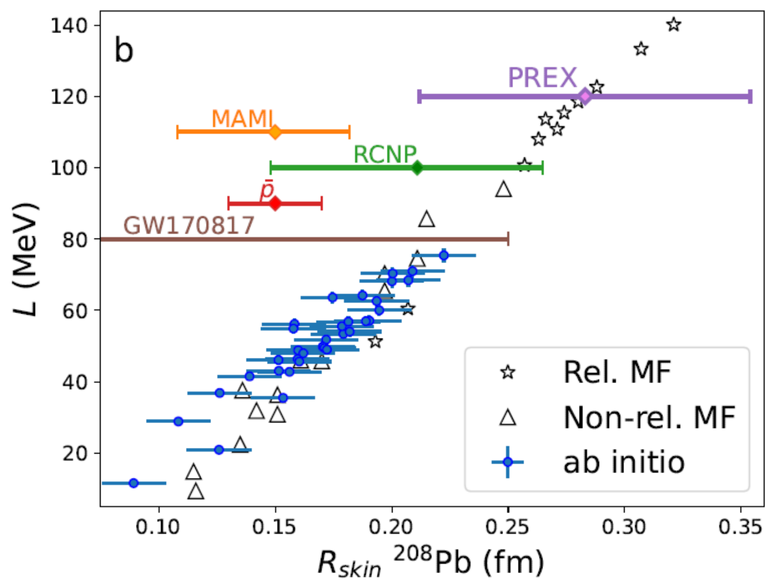

Revised Extended Data Fig. 7b

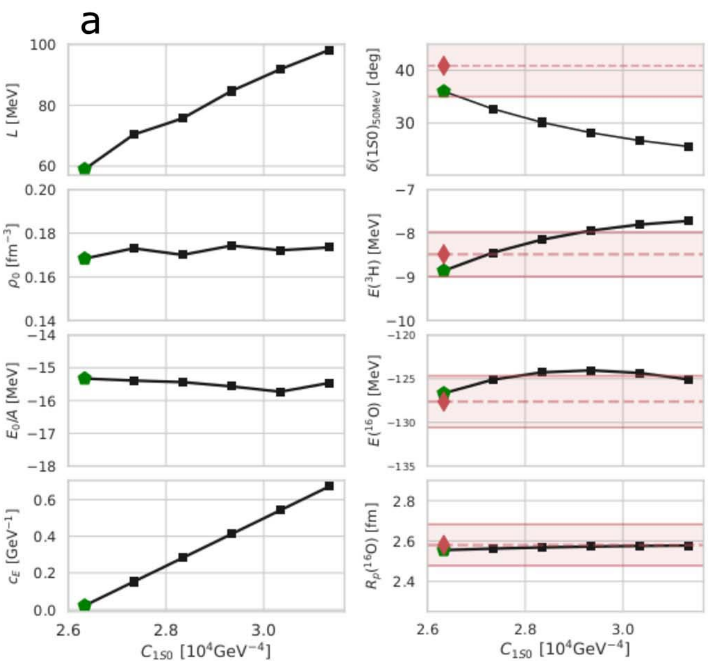

Original Extended Data Fig. 8a

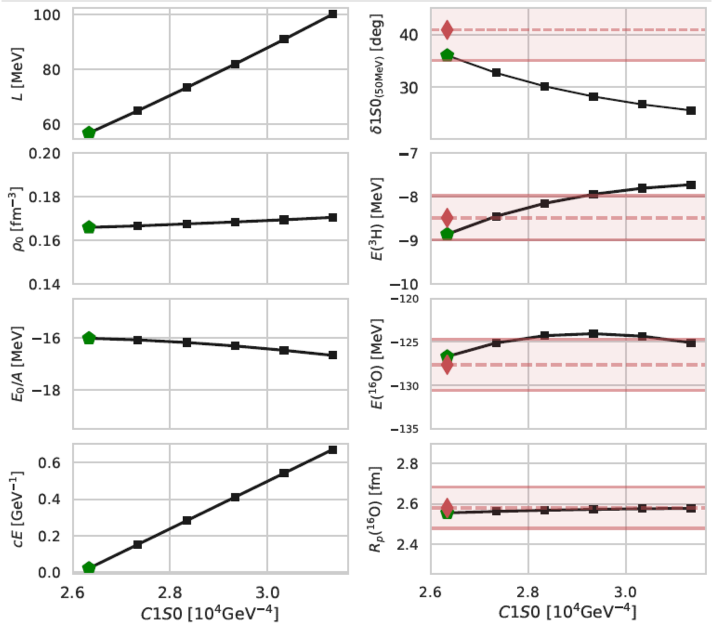

Revised Extended Data Fig. 8a

Nuclear matter properties			
Observable	median	68% CR	90% CR
E_0/A	-15.2	$[-16.3, -13.9]$	$[-17.1, -13.4]$
ρ_0	0.163	[0.147, 0.176]	[0.140, 0.186]
S	29.1	[26.8, 31.4]	[25.433.0]
L	50.5	[36.6, 66.3]	[23.6, 74.8]
K	264	[219, 300]	[202, 336]
Neutron skins			
Observable	median	68% CR	90% CR
$R_{skin}(^{48}Ca)$	0.164	[0.141, 0.187]	[0.123, 0.199]
$R_{skin}(^{208}Pb)$	0.171	[0.139, 0.200]	[0.120, 0.221]

Original Extended Data Table 2

Nuclear matter properties			
Observable	median	68% CR	90% CR
E_0/A	-16.9	$[-17.9, -15.4]$	$[-19.1, -14.9]$
ρ_0	0.167	[0.150, 0.181]	[0.142, 0.194]
S	31.1	[29.1, 33.2]	[27.6, 34.6]
L	52.7	[38.3, 68.5]	[23.9, 76.2]
K	287	[242, 331]	[216, 362]
Neutron skins			
Observable	median	68% CR	90% CR
$R_{skin}(^{48}Ca)$	0.164	[0.141, 0.187]	[0.123, 0.199]
$R_{skin}(^{208}Pb)$	0.171	[0.139, 0.200]	[0.120, 0.221]

Revised Extended Data Table 2
